# Population response to the risk of vector-borne diseases: lessons learned from socio-behavioural research during large-scale outbreaks

**DOI:** 10.3134/ehtj.09.006

**Published:** 2009-07-31

**Authors:** M Setbon, J Raude

**Affiliations:** 1 National Center for Scientific Research, Institute of Labour Economics and Industrial Sociology, Aix-en-provence, France; 2 EHESP School of Public Health, Center for Research on Risk and Regulation, Paris, France

## Abstract

Vector-borne infectious diseases, such as malaria, dengue, chikungunya, and West Nile fevers are increasingly identified as major global human health threats in developing and developed countries. The success or failure of vector control rests mainly on the nature and scale of the behavioural response of exposed populations. Large-scale adoption of recommended protective behaviour represents a critical challenge that cannot be addressed without a better understanding of how individuals perceive and react to the risk of infection. Recently, French overseas territories faced large-scale outbreaks: an epidemic of chikungunya fever in La Re′ union and Mayotte (2005–2006) and four successive outbreaks of dengue fever in one Caribbean island, Martinique (1995–2007). To assess how these populations perceived and responded to the risk, and how the nature and scale of protection affected their clinical status, socio-epidemiological surveys were conducted on each island during the outbreaks. These surveys address three crucial and interconnected questions relevant to the period after persons infected by the virus were identified: which factors shape the risk of acquiring disease? Which socio- demographic characteristics and living conditions induce a higher likelihood of infection? What is the impact of risk perception on protective behaviours adopted against mosquito bites? Grounded on the results of these surveys, a general framework is proposed to help draw out the knowledge needed to reveal the factors associated with higher probability of infection as an outbreak emerges. The lessons learnt can inform health authorities’ efforts to improve risk communication programmes, both in terms of the target and content of messages, so as to explore new strategies for ensuring sustainable protective behaviour. The authors compare three epidemics of vector-borne diseases to elucidate psychosocial factors that determine how populations perceive and respond to the risk of infectious disease.

## Introduction

The risk of large-scale epidemics has returned in the early 21st century. The threat of new and re-emerging infectious diseases is increasingly recognised as one of the more likely and potentially devastating events that humanity could face in the coming decades.^1^ Despite their disproportionate share of disease burden in terms of morbidity and mortality in some parts of the world, communicable infectious diseases are not only the concern of developing countries.^2^ Malaria, HIV (Human Immunodeficiency Virus)/AIDS, tuberculosis, dengue fever, and West Nile fever still affect a large number of Asian, American, and European countries. In addition, over the past 2 or 3 decades, western and developed countries have faced growing threats from vector-borne emerging infectious diseases (EID) such as chikungunya fever, dengue fever, West Nile fever, and Lyme disease.^3^–^5^ Urbanisation, climate change, globalisation, migration, and other socio-ecological factors are recognised as primary conditions that could lead to possible large-scale outbreaks of arboviral diseases.^6^–^8^ For most diseases caused by these viruses, there is no available treatment or vaccine, and populations in developed coun-tries have not experienced them at epidemic levels. Even though no large-scale epidemics have occurred yet in Europe, recent alerts have brought to light the risk of mosquito-borne infectious diseases.^9^, ^10^ For example, a major epidemic of chikungunya fever was recently narrowly avoided in Italy.^11^

To date, numerous relevant epidemiological, virological, and clinical data have been collected to identify the main features that could characterize an EID and help to fight against it. Nevertheless, whatever the availability of scientific knowledge and the quality of its implementation into public health programmes, successful response rests, to a large extent, on the relevance of the behaviours adopted by the exposed populations. In other words, the more accurately the public evaluates the risk and complies with public health recommendations, the smaller the amplitude of the epidemic and its harmful consequences tend to be. From this perspective, understanding how and why the public perceives and reacts to the threat of an EID—what determines the pattern and distribution of protective behaviours— represents one of the most crucial challenges both in terms of scientific knowledge and in terms of health and socioeconomic impacts.

In recent decades, an important and consistent body of work has developed to examine how people perceive diseases and the risk of infection. This research aims to help identify the factors that explain and predict health behaviour.^12^, ^13^ Integrative models of cognitive, affective, and social determinants of health behaviour have been proposed to describe and predict the processes involved in the self-regulation of health threats.^14^, ^15^ More recently, the psychometric approach of Slovic and colleagues^16^–^18^ has provided a wide array of useful information as to the manner in which people judge and react to technological and environmental threats, as well as the cognitive and perceptual processes they undertake in a hazardous exposure setting. Paradoxically, little is known about changes in perception and behaviour induced by sudden large-scale epidemics in developed countries. This gap in understanding could be explained by the fact that such epidemics have been absent from developed countries for a long time. Much of the research concerning infectious risk has been carried out on HIV/AIDS or on more benign/frequent diseases, such as seasonal influenza or Lyme disease.^19^

According to the socio-behavioural literature, people generally perceive and react to health threats according to complex processes in which earlier experiences, information, attitudes, beliefs, and emotions contribute to produce a judgment or an intention to act and, under some conditions, result in behavioural change. Identification of the factors that determine individual perceptions and adoption of protective actions, as well as their sociological distribution, would help direct the aims, content, and targets of risk management and communication.^20^ Here, we present the experience that we have drawn from surveys carried out during both epidemic and endemic vector-borne disease events, and we address questions concerning (1) the social distribution of arboviral infections related to large-scale exposure; (2) the psycho-cognitive factors driving perceptions of risk and their relationship with behaviour change; and (3) the lessons that could be learnt from the range of results obtained in different contexts.

## The overseas French territories experience

Beyond the few recent outbreaks of EIDs, such as severe acute respiratory syndrome or bovine spongiform encephalopathy, which have affected many developed countries, France has recently faced large outbreaks in its overseas territories: an epidemic of chikungunya fever in La Réunion^21^ and Mayotte (2005–2006), and four successive outbreaks of dengue fever in the Caribbean island, Martinique (1995–2007). The interest in studying these outbreaks as models lies in their having three key elements in common. First, they occurred in communities benefiting from the same standard of healthcare facilities and modern tools of disease surveillance and monitoring (epidemiological staff, heath administration, networks of communication, etcetera), as in developed countries of Western Europe, Asia, and America. Second, chikungunya and dengue fevers are both mosquito-borne diseases: the first is transmitted by the mosquito *Aedes albopictus* and the second by *Aedes aegypti*—two species of mosquitoes that are seen frequently in tropical countries and that are now present in western latitudes.^22^ Third, once the outbreaks were identified and the population informed of the nature and the cause of disease spread (mosquito bites), the primary message in terms of protective behaviour that was disseminated by heath authorities and amplified by the media was simple: ‘You have to destroy mosquitoes and protect yourself from their bites.’ Let us underline that mosquitoes are very familiar to the populations, as they have been present in these countries for a long time, hence exposure to mosquito bites is common (Table 1).

**Table 1:** The context and design of the three surveys

*Affected island*	*La Réunion*	*Mayotte*	*Martinique*
Location	Indian Ocean	Indian Ocean	Caribbean Sea
Population (inhabitants)	750,000	170,000	399,000
Date of the outbreak (number)	2005–2006 (1)	2005–2006 (1)	1995–2007 (4)
Epidemic/endemic	Epidemic	Epidemic	Endemic+epidemic
Main vector	Aedes albopictus	Aedes albopictus	Aedes aegypti
Virus	Chikungunya	Chikungunya	Dengue
% Population infected	38	38	20
Date of survey	May 2006	December 2006	June 2007
Sample size	*N=*1035	N=888	*N=*1001
Administration method	Telephone	Telephone	Face to face
Status identification	Questionnaire	Questionnaire+serology	Questionnaire

**Figure 1:**
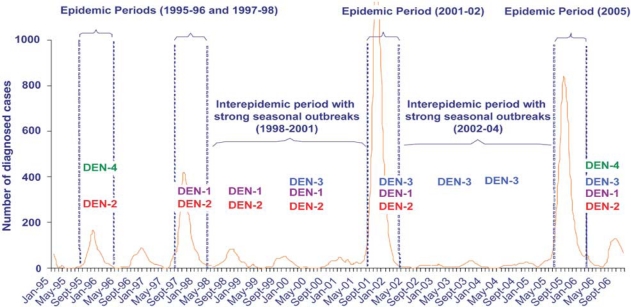
Epidemic curve of dengue fever in Martinique between 1995 and 2006 (Source: CIRE Antilles-Guyane).

Despite intensive communication and pest control campaigns, a large part (260,000 or 38%) of the population of La Réunion was infected by chikungunya virus from March 2005 to June 2006,^23^ and the same proportion of the Mayotte population was infected by the virus during the same period.^24^ Approximately 20% of residents in Martinique were infected (at least once) by one of the four types of dengue virus identified between 1995 and 2007.^25^ Figure 1 illustrates the epidemic curve of dengue epidemics in Martinique and the four strains identified from 1995 to 2006. Nevertheless, it should be noted that the chikungunya outbreaks that had previously occurred in developing countries led to considerably higher rates of infection: Kenya was stricken by an intense epidemic of chikungunya fever beginning in January 2004, particularly in coastal regions. A seroprevalence survey indicated that 75% of the population of the Lamu and Mombasa regions was infected by chikungunya virus.^26^ In Grand Comoros, where a chikungunya epidemic began in January 2005, the attack rate reached 63%.^27^

We propose the hypothesis that the differences in infection rates between these outbreaks and the events in the La Réunion/Mayotte islands were because of the effect of communication and pest control campaigns. Presently, this remains a hypothesis that can be verified only through the development of mathematical models. (To verify this hypothesis, we launched a new research initiative presently in progress (2009) whose objectives are to assess retrospectively: the epidemic potential of the disease, how it could have developed without interventions, the efficacy of interventions, and the role of individual behaviours in shaping the epidemic.) However, as rates of infection varied in La Réunion and Mayotte from 10 to 70%, depending on the region, the main question is this: What factors could explain the fact that some communities saw significantly higher rates of infection than others in a population frequently and uniformly exposed to mosquito bites? The case of dengue fever in Martinique, although somewhat different because it was characterized by successive outbreaks within a long-lasting endemic situation, poses the same question.

To explore this question, three socio-epidemiological surveys, one on each island, were conducted on representative samples of the islands’ adult population (2006–2007). The main objective in each case was to identify the socio-demographic distribution of the incidence in relation to the psycho-cognitive, behavioural, and environmental data collected to determine which factors could explain the disparities in the prevalence of chikungunya/dengue fever disease within each island. Here, we will summarise the main results from a series of publications in which all details about the methods, results, and discussions have been presented.^28^–^30^ Finally, we will propose an overall analytic approach focused on the framework driving the arboviral outbreaks and on the lessons that could be learnt for future outbreaks in developed countries.

### The determinants of vulnerability to arbovirus infection

All three surveys were based on a questionnaire that included a series of items about the experience of the disease (if contracted), knowledge of the nature and cause of the disease, perception of the risk and of effectiveness of protective measures, adoption of protective behaviour, beliefs and attitudes towards the illness, and socio-demographic characteristics. The surveys were conducted either by phone or face-to-face (Table 1). To identify those who were infected on the three islands (the dependent variable to be explained), we used two kinds of tools. First, the answer provided by individuals to the question ‘Did you get or do you now have the chikungunya (or dengue) fever?’—a question asked in the three surveys. Second, and only in the case of Mayotte, we used a serological survey through blood sample collection.

The final results from the questionnaire data on the percentage of the population infected were: 38% of the population in La Réunion, as in Mayotte, contracted chikungunya fever and 20% of the population in Martinique contracted dengue fever. These results seemed congruent with the results of seroprevalence surveys done later in La Réunion,^23^ with serological survey results in Mayotte, as well as with the estimates calculated in Martinique.^31^

Subsequently, statistical analyses were performed to identify the factors significantly associated with the risk of contracting the disease. Bivariate analyses indicated that in La Réunion, the people more vulnerable to infection with chikungunya virus were those who were socially deprived, that is, less educated and with a lower income, those born on the island, and those living in separate housing units with a garden (Table 2). On the other hand, in terms of perception and behaviour, reduced infection risk was associated with perceiving the controllability of the disease, the usefulness of personal protection, and frequently using mosquito-repellent sprays (Table 3). This finding was roughly the same in Mayotte, where the population had the same attack rate from the infection by the end of the epidemic. The same socio-demographic and psycho-cognitive factors were found to be significantly associated with chikungunya fever in Mayotte, with the exception of place of origin (migrants were more likely to be infected compared with the native population), and a lack of impact from individual protective behaviours. It should be noted here that the majority of migrants in La Réunion originate from European territories, whereas those in Mayotte come from poorer territories (mainly from the Comoros Islands and Madagascar).

**Table 2:** Prevalence of chikungunya/dengue fever by socio-demographic variables

*Variables*	*La Reunion percentage*	*Significance level*	*Mayotte percentage*	*Significance level*	*Martinique percentage*	*Significance level*
*Place of birth*						
Reunion/Mayotte/Martinique	46.1	< 0.001	29.3	<0.001	18.4	<0.01
Other location	23.9		52.8		30.9	
*Type of housing*						
Individual house	47.0	<0.001	39.3	NS	22.1	<0.01
Collective	19.4		30.3		15.9	
*Education*						
No formal education	49.1		41.5		12.8	
Some high school	46.5	<0.001	29.7	<0.05	19.8	<0.01
High school graduate	37.2		28.2		19.6	
Some college	30.0		25.0		27.8	
*Occupation*						
Student	35.6		34.4		17.4	
Housewife	44.6		37.5		15.3	
Unemployed	47.0	NS	42.6	NS	19.0	<0.05
Employee/independent worker	41.1		33.9		23.9	
Retired	39.4		29.4		14.1	
*Household size*						
1–2	40.1		37.2		20.9	
3–4	40.3	NS	46.7	<0.001	19.4	NS
5–6	46.3		41.3		18.1	
≥7	45.5		32.7		6.3	
Total	41.6		39.0		19.4	

Abbreviation: NS, non-significant (*P*>0.05).

**Table 3:** Prevalence of chikungunya/dengue fever by psycho-cognitive variables

*Variables*	*La Réunion percentage*	*Significance level.*	*Mayotte percentage*	*Significance level*	*Martinique percentage*	*Significance level*
*Perceived controllability*						
Agree	37.0	<0.001	36.6	<0.001	19.7	NS
Disagree	54.0		51.0		19.7	
*Perceived effectiveness of protection*						
Agree	38.7	<0.01	38.4	N.S.	16.4	NS
Disagree	49.4		45.2		20.2	
*Perceived route of infection: mosquitoes*						
Agree	38.7	<0.01	38.2	NS	19.7	NS
Disagree	49.1		40.6		27.1	
*Perceived route of infection: air*						
Agree	50.7	<0.001	39.6	NS	15.1	<0.05
Disagree	35.2		37.7		22.6	
*Perceived route of infection: body contact*						
Agree	48.4	<0.001	42.4	NS	17.3	<0.05
Disagree	37.7		37.1		23.3	
*Use of repellents*						
Agree	35.8	<0.01	MD	MD	16.9	NS
Disagree	45.9		MD		19.7	
Total	41.6		39.0		19.4	

Abbreviations: MD, missing data; NS, non-significant (*P*>0.05).

In Martinique, where dengue fever was endemic and four outbreaks had occurred since 1995, the finding was surprisingly quite different. Analysis of the empirical data gave opposite results, with the notable exception of housing conditions. The socio-demographic factors associated with infection were: upper-class status, high level of education and income, better knowledge of dengue risk, living in a private house with a garden, and having been born in metropolitan France. With respect to psycho-cognitive variables, no risk factors were identified apart from a fatalistic attitude,^32^ which was significantly and paradoxically associated with a slightly lower probability of contracting dengue fever.

Finally, we conducted a series of logistic regression analyses of the data collected in each island. This produced statistical results that were not substantially different from the pattern of correlations found in the bivariate analysis.

## Arboviral outbreaks: a general framework for EID

Arboviral diseases are representative of communicable infectious disease outbreaks in terms of the transmission process involved and the knowledge needed to respond to an EID. The general framework starts with an invisible change in the environment of the populations concerned. In the case of an arboviral disease outbreak, the change would be denoted by an acquired competency of a vector to become a carrier and a transmitter of a virus. Given that mosquitoes have been present for a long time and are perceived as harmless by the public in affected areas, recognising them as the causal agent takes time, and this new perception is not uniformly shared. So, when a new infection is emerging, the perceived risk of contracting it through mosquito bites is hidden by the lack of a noticeable change in the familiar environment of the population. This represents the starting point common to most infectious disease outbreaks. After a certain amount of time, despite the fact that the causal agent and its routes of transmission are well and publicly recognised, a subset of the public continues to perceive the risk of infection as unreal, inconsistent, or too costly to prevent. In this general scenario, the outcome of the out-break is made apparent through the spread of the infection among the exposed population. Identifying the main factors that drive the spread, their interdependencies, and their relative weight represent both a generic objective for any infectious disease outbreak response and a key piece of information that can be used to adapt and improve infection control.

In large-scale outbreaks of arboviral or other infectious diseases, the general research objective is to uncover the relationships between the epidemiology of the outbreak and socio- or psycho-behavioural factors that characterise the exposed population. Apart from genetic susceptibility, which is a parameter generally inaccessible to investigators, the relevant knowledge that can be gathered consists of identifying three major dimensions that determine the probability distribution of the disease: (1) A set of socio-demographic factors that represent existing living conditions described with an objective measure, such as socio-economic status, housing, or socio-cultural background.^33^, ^34^ (2) The level of exposure that could be relatively easy to assess depending on whether the agent is visible and widespread, or invisible and unevenly distributed geographically. In the case of an arboviral disease outbreak, the exposure to the viral agent is made visible thanks to its carriage by mosquitoes. The exposure to mosquito bites in the three islands studied, although difficult to assess accurately, is assumed to be uniform across the population given the wide spatial distribution of the vector and also given the shared housing conditions. (3) The level of individual protection that could reduce the effects of the natural exposure and seems to be the primary factor that can discriminate between infected and uninfected individuals. Thus, when differences are observed in socio-cognitive factors and are associated with behavioural consequences, they are expected to play a causal role in the final outcome, which is defined as ‘infected’ or ‘infection-free’ individuals.^35^ In the events we studied, that was clearly the case only in La Réunion, where a perception of having behavioural control over the risk and a belief in the usefulness of personal protection measures were significantly associated with use of repellent sprays, and finally with having infection-free status.

The same framework could explain the case of dengue fever in Martinique, even though the results seemed to be somewhat different. It was shown in the survey that despite a good, shared understanding within the population regarding dengue fever and the means of protection against the disease, the vast majority seemed reluctant to take the corresponding behavioural precautions. This established fact is congruent with an absence of psycho-cognitive factors associated with better protective behaviour, a condition necessary to reduce the risk of infection. In other words, as no significant difference in the level of actual individual protection was found, the distribution of dengue fever, which varies between socio-demographic groups, would be primarily determined by the distribution of the natural exposure modified by collective protection measures against mosquito bites (pest and larval control campaigns). This hypothesis is consistent with the surprising and uncommon socio-demographic variables characterising the group most likely to be infected in La Martinique. However, further research is needed to confirm this hypothesis, particularly by introducing data about the geographical allocation of pest control interventions and some evaluation of their impact.

We must note that there is a strong interdependency among socio-demographic and psycho-cognitive dimensions in risk perception and behavioural changes. Although much research in the past has shown the impact of social factors on health, the associations found here between the risk of infection and certain socio-demographic variables such as sex, age or education would more likely be seen as markers of infection or confounding factors than as causal factors.

## Some lessons for future large-scale infectious disease outbreaks

Large-scale outbreaks of (re-)emerging infectious diseases would be better and more rapidly controlled if their distribution were understood through surveys aiming to identify the factors corresponding to the three dimensions described above. The level of protection adopted by the exposed individuals represents not only the main determinant of the clinical outcome but also the main factor that can be easily influenced by new infection control programmes. From this perspective, the first major lesson drawn from the results of these surveys concerns the distribution of risk perception.^36^ Individual levels of risk perception represent an important point to investigate through its key variables: perceived likelihood of infection, perceived severity of the disease, and perceived self-efficacy.^37^ When the threat is perceived to be serious, as shown by a high perceived susceptibility and severity, it becomes a condition that can lead (or not) to an effective change in behaviour. The perceived threat seems to be different in the case of chikungunya fever outbreaks compared with the case of dengue fever epidemic/endemic situations studied. In the first case, the protective behaviour indicated by the health status was found to be associated with perceived controllability of the disease and perceived usefulness of personal protection, an outcome of risk perception.^38^ In the case of dengue fever, the distribution of risk perception does not seem to be associated either with protective behaviour or with health status. Whatever the role and impact of risk perception, there is a need for cognitive and perception- oriented studies of the exposed population in large-scale infectious outbreaks.

The second lesson relates to the adoption of protective behaviour in such events. In the scientific literature on risk, the link between risk perception and protective behaviour has been extensively questioned: although often identified, it rarely seems to be very significant and is sometimes inconsistent.^39^ Other factors can drive actions related to risk reduction: ‘perceived barriers’ and ‘perceived benefits’ were identified as playing an important role in the translation of the perceived risk to an actual protective behaviour.^40^ This relationship would also be influenced by specific conditions surrounding the problem (large-scale or rare epidemic), the nature of the threat (expected or actual, new or old, epidemic or endemic, severe or benign, etcetera), the design of the survey (longitudinal or cross-sectional), and the questions asked to measure the ‘perceived risk.’ The outcome of risk perception may have different manifestations. It could be expressed through a judgement call, an intention to change behaviour, or an actual (or the absence of) behavioural change. Note that the three cases of large-scale outbreaks studied here represent a rare opportunity in the socio-behavioural literature to define concretely the relationships between risk perception, behaviour, and a dichotomous clinical outcome.

The third important lesson concerns the sustainability of adherence to a protective behaviour, which is undoubtedly affected by the length of the outbreak. After a certain amount of time, when the threat becomes better understood and familiar, the fear triggered by the novelty of the threat is superseded by a process of normalisation, posing the question of how high-risk perception and protective behaviours can be maintained sustainably in endemic contexts. This phenomenon of relapse, well identified in long-term epidemics of disease such as HIV/AIDS,^41^ enables one to explain, at least partly, the paradoxical findings in Martinique. Despite a good understanding of the disease and a consensus on the efficacy of the main protective tools available, people do not use them enough to be protected. Thus, endemic disease can be viewed as a particularly challenging context for successfully implementing risk-reduction strategies because of the difficulty of ensuring sustainable protection. The challenge is to translate this finding into relevant risk management and communication approaches.

## Proposed outbreak management strategies

Two different strategic options can be proposed for managing the risk of long-lasting outbreaks of disease, such as dengue fever. The first option is to attempt to convince people to protect themselves as long as the virus is circulating, regardless of the context, that is, endemic or epidemic disease. Our results show that this objective seems very difficult to achieve and is probably unrealistic in the case of permanent or long-term health threats. Study of sustainable health-protective behaviour indicates that numerous difficulties make this aim unachievable.^13^ However, an alternative option would be to adopt a flexible and targeted strategy: compliance to an effective protection measure would be asked in accordance with the level of viral exposure. The aim would be to achieve behavioural change limited to a temporary, but real protection, when the level of circulating virus is critical, as opposed to seeking an inaccessible and permanent degree of protection that is considered too costly for the vast majority of the population. This strategy would permit one to take into consideration the ratio of the two main components of risk reduction: benefits expected from a temporary protection and the level of exposure. In the case of a mosquito-borne virus, the higher the level of exposure the lower the probability of remaining healthy without adopting intensive, daily protection measures. In contrast, the benefits of having permanent protection during low levels of exposure are poor, because the risk of infection through mosquito bites is minor. This flexible strategy related to exposure enables one to better address the low capacity to sustain long-term and efficient individual protection in a cyclical context. The experience of the chikungunya outbreak in La Réunion indicates that the proportion of the population who protected themselves effectively during a short period reduced their risk of infection. However, two conditions are needed for this strategy to succeed in an endemic context: one is the capacity to monitor and predict the arrival of a new outbreak,^42^ another is the ability to rapidly translate the alarm into a strong and convincing communication, as in the case of hurricanes, floods, heat waves, etcetera. From our viewpoint, protection against vector- borne diseases, such as chikungunya or dengue fevers, should benefit considerably from an exposure-related strategy, that is, modulating the fight against mosquitoes according to the level of exposure to the virus. This would improve the efficiency of protection measures and ultimately reduce the rate of infection.

In conclusion, if elucidating the role and impact of risk perception in large-scale infectious disease outbreaks is recognised as useful, the relevance of such psycho-socio-epidemiological studies would be improved by following four major avenues: (1) organising timely surveys to reveal the distribution of risk perception parameters and how they relate to intended or actual behaviour: the development of targeted programs for better control of the outbreak, implemented through risk communication and health education campaigns, would be grounded on their results; (2) assessing the impact of public health campaigns on behaviour change through evaluation of community participation to reduce mosquito breeding sites; (3) monitoring the perceived risk of the exposed population with longitudinal surveys when the outbreak shows signs of becoming endemic; (4) connecting and integrating the data collected in these surveys with serological, entomological, and geographical data, which would then be entered into models aiming to forecast the course of an epidemic.
